# Pectinase secreted by psychrotolerant fungi: identification, molecular characterization and heterologous expression of a cold-active polygalacturonase from *Tetracladium* sp.

**DOI:** 10.1186/s12934-019-1092-2

**Published:** 2019-03-07

**Authors:** Mario Carrasco, Juan Manuel Rozas, Jennifer Alcaíno, Víctor Cifuentes, Marcelo Baeza

**Affiliations:** 1 Innocacold S.A, Santiago, Chile; 2 Laboratorio de Genética, Departamento de Ciencias Ecológicas, Facultad de Ciencias, Universidad de Chile, Las Palmeras 3425, Casilla 653, Santiago, Chile

## Abstract

**Background:**

Pectinolytic enzymes, which are used in several industries, especially in the clarification process during wine and fruit juice production, represent approximately 10% of the global enzyme market. To prevent the proliferation of undesired microorganisms, to retain labile and volatile flavor compounds, and to save energy, the current trend is to perform this process at low temperatures. However, the commercially available pectinases are highly active at temperatures approximately 50 °C and poorly active at temperatures below 35 °C, which is the reason why there is a constant search for cold-active pectinases. In preliminary studies, pectinolytic activity was detected in cold-adapted yeasts and yeast-like microorganisms isolated from Antarctica. The aim of the present work was to characterize pectinases secreted by these microorganisms and to express the best candidate in *Pichia pastoris*.

**Results:**

Degradation of pectin by extracellular protein extracellular extracts obtained from 12 yeast cultures were assayed in plates at 4 °C to 37 °C and pH from 5.4 to 7.0, obtaining positive results in samples obtained from *Dioszegia* sp., *Phenoliferia glacialis* and *Tetracladium* sp. An enzyme was purified from *Tetracladium* sp., analyzed by peptide mass fingerprinting and compared to genome and transcriptome data from the same microorganism. Thus, the encoding gene was identified corresponding to a polygalacturonase-encoding gene. The enzyme was expressed in *Pichia pastoris*, and the recombinant polygalacturonase displayed higher activity at 15 °C than a mesophilic counterpart.

**Conclusions:**

Extracellular pectinase activity was found in three yeast and yeast-like microorganisms from which the highest activity was displayed by *Tetracladium* sp., and the enzyme was identified as a polygalacturonase. The recombinant polygalacturonase produced in *P. pastoris* showed high activity at 15 °C, representing an attractive candidate to be applied in clarification processes in the production of fermented beverages and fruit juices.

**Electronic supplementary material:**

The online version of this article (10.1186/s12934-019-1092-2) contains supplementary material, which is available to authorized users.

## Background

Currently, there is a high demand for enzymes used in several industrial applications for food, detergent, paper, textile and synthesis of organic compounds because they are highly efficient and environmentally friendly [1–3]. Additionally, these enzymes constitute a well-established global market projected to reach US$6.3 billion in 2021 [1, 4]. The current trend is to use cold-adapted or cold-active enzymes to decrease the temperature of the industrial processes, allowing energy savings and diminishing their carbon footprint and to manufacture products with better performance at ambient or lower temperatures [5–7]. Approximately 10% of the enzyme market is represented by pectinolytic enzymes [8, 9], which are used in the wine, food, paper and textile industries [9, 10]. The reduction of cloudiness and bitterness of fruit juices and grape must in the juice and wine industries is performed at low temperatures (≤ 15 °C) to prevent the proliferation of undesired microorganisms, to retain the labile and volatile flavor compounds, and to save energy [7, 9]. These requirements have led to the search for pectinases with high performance at lower temperatures but also at low pH, as the pH of fruit juices and grape must be in the range from 2.5 to 3.5 [11]. Currently, the industrially available pectinases are obtained from mesophilic filamentous fungi, mainly from *Aspergillus* species; however, they work poorly at temperatures ≤ 35 °C [12]. Thus, cold-active enzymes have higher enzymatic activities at lower temperatures than their mesophilic counterparts [13].

Microorganisms that thrive in cold environments have evolved several adaptations to live under this condition, including the synthesis of cold-active enzymes [14–17]. In particular, cold-adapted fungi secrete cold-active enzymes that hydrolyze the complex compounds available in the environment to use as nutrients [18–21]. The production of pectinases has been reported in cold-adapted bacteria and fungi [11, 22–36]; however, in most of these studies no purification or biochemical characterization of the enzyme were performed. Pectinolytic enzymes, or pectinases, are classified according to their mode of action and to their substrate: polygalacturonases, which are subclassified as endo-polygalacturonases (E.C. 3.2.1.15) and exo-polygalacturonases (E.C. 3.2.1.67); lyases, which are classified into pectate lyases (E.C. 4.2.2.9 and EC. 4.2.2.2) or pectin lyases (E.C. 4.2.2.10); and pectin methylesterases (E.C. 3.1.1.11). It is recommended the use of a combination of different kind of pectinases that degrade different parts of the polymer, to achieve a maximal degradation of pectin in various raw materials [37]. Bacteria produce alkaline pectinases, most frequently polygalacturonases and pectate lyases [38]. In fungi, the production of acidic pectinases has been described, mainly exo- and endo- polygalacturonases [38]. The most frequently used pectinases in industry are the polygalacturonases, which belong to glycosyl hydrolase family 28 (GH28). Although there have been attempts to isolate cold-active pectinases from different sources, to the best of our knowledge, there are no commercially available cold-active pectinases.

In previous work, pectinolytic activity was detected in 12 yeasts and yeast-like microorganisms isolated from soils of King George Island in the sub-Antarctic region [22]. In this work, these microorganisms were further studied to characterize the pectinases secreted by them. Secretion of pectinase was confirmed in three of them, and among these, *Tetracladium* sp. showed the highest pectinase activity. The enzyme from *Tetracladium* sp. was purified and analyzed by peptide mass fingerprinting. The peptide sequences were compared to genome and transcriptome data, and the gene, which encodes a polygalacturonase, was identified. The gene was expressed in *Pichia pastoris*, and the recombinant polygalacturonase displayed higher activity at 15 °C than a mesophilic counterpart.

## Methods

### Strains, plasmid and growth conditions

Yeast strains used in this work and their relevant characteristics are listed in Table 1, wich were isolated, identified and characterized by our group from soil samples of King George Island at the sub-Antarctic [22]. The yeasts were routinely cultivated in yeast-malt medium (YM): 3 g L^−1^ yeast extract, 3 g L^−1^ malt extract and 5 g L^−1^ peptone, supplemented with 10 g L^−1^ of glucose or 10 g L^−1^ of pectin (pectin of citrus peel, Sigma-Aldrich Corporation, MO, USA). *E. coli* strains were cultivated in lysogeny broth medium (LB): 10 g L^−1^ tryptone, 5 g L^−1^ yeast extract and 5 g L^−1^ NaCl, supplemented with 1 g L^−1^ glucose; ampicillin was added at 100 mg ml^−1^ when necessary. Synthetic defined medium (SD: 20 g L^−1^ glucose and 6.7 g L^−1^ yeast nitrogen base) was used for selection of recombinant *P. pastoris* strains. Buffered complex medium (BCM: 10 g L^−1^ yeast extract, 20 g L^−1^ peptone, 13.4 g L^−1^ yeast nitrogen base, 0.004 g L^−1^ biotin, 10 g L^−1^ glycerol and 100 mM potassium phosphate buffer pH 6.0), supplemented with 10 g L^−1^ glycerol or 0.5% methanol when necessary, was used for gene expression in *P. pastoris*. For semisolid-media, 15 g L^−1^ of agar was used.

**Table 1:** Microorganism, plasmids and oligonucleotides used in this work

Microorganism/plasmid/oligonucleotide	Description	References
Yeast/yeast-like		
*Tetracladium* sp.	30 °C^a^	[22]
*Dioszegia* sp.	15 °C^a^	[22]
*Rhodotorula glacialis (Phenoliferia glacialis)* (T8Rg)	22 °C^a^	[22]
*Leuconeurospora* sp. T17Cd1	15 °C^a^	[22]
*Leucosporidiella fragaria* (*Leucosporidium fragarium)*	22 °C^a^	[22]
*Metschnikowia* sp.	10 °C^a^	[22]
*Mrakia psychrophila*	10 °C^a^	[22]
*Mrakia robertii*	15 °C^a^	[22]
*Rhodotorula glacialis (Phenoliferia glacialis)* (T11Rs)	22 °C^a^	[22]
*Rhodotorula* (*Cystobasidium*) *laryngis*	30 °C^a^	[22]
*Sporidiobolus salmonicolor*	22 °C^a^	[22]
*Wickerhamomyces anomalus*	30 °C^a^	[22]
PichiaPink strain 4	*P. pastoris* strain: *ade2, prb1, pep4*	Invitrogen
PichiaPink strain 2	*P. pastoris* strain: *ade2, pep4*	Invitrogen
Bacteria		
*Escherichia coli* Top10	F-mcrA Δ(mrr-hsdRMS-mcrBC) Φ80lacZΔM15 Δ lacX74 recA1 araD139 Δ(araleu)7697 galU galK rpsL (StrR) endA1 nupG	Thermo Fisher
Plasmids		
pPinkα-HC	*P. pastoris* integrating vector for high-copy expression of a secreted protein	Invitrogen
Oligonucleotides		
Pectfw	5′-GCACCTACAGTCTCATCATTG-3′	This work
Pectrev	5′-GCAGGAAGCAGGGGATGGGAA-3′	This work

^a^Optimal temperature for growth. The current taxonomic classification is given in parentheses

### Molecular and biochemical methods

Standard molecular and biochemical procedures, such as plasmid DNA purification, digestion with restriction enzymes, cloning procedures, PCR assays, SDS-PAGE, protein quantification and electrotransformation, were performed according to standard protocols [39]. Protein quantification was made using the BCA Kit Assay (Thermo Scientific, IL, USA). Plasmid and genomic DNAs were purified using the GeneJet Plasmid Miniprep Kit (Thermo Scientific, Waltham, MA, USA) and the Wizard Genomic DNA Purification kit (Promega, WI, USA) according to manufacturer’s instructions.

### Extraction and purification of extracellular proteins

Yeast cultures (100–300 mL) at the initial stationary phase of growth were centrifuged at 7000×*g* for 10 min at 4 °C and filtered through sterile 0.45-μm pore size polyvinylidene fluoride membrane filters (Millipore, Billerica, MA, USA). Ammonium sulfate was added to the cell-free supernatants to reach 80% of saturation to obtain total proteins or in the case of protein fractioning, to reach increasing saturation from 20 to 80%. Samples were incubated at 4 °C for 2 h and centrifuged at 10,000×*g* for 15 min at 4 °C. The protein pellets were suspended in 2–4 mL of 20 mM potassium phosphate and 150 mM NaCl pH 7.0, and samples were desalted using a HiTrap Desalting column (GE, Schenectady, Nueva York, USA) in AKTA Prime purification equipment (GE). For ion exchange protein purification, the protein sample was loaded onto a DEAE-Sephadex column equilibrated with 50 mM Tris–HCl pH 7.0, proteins were eluted using a NaCl gradient from 0 to 200 mM as the mobile phase at 0.5 mL min^−1^, and 1 mL fractions were collected. For gel filtration purification, 0.2 mL of concentrated protein extracellular extracts were loaded onto a Superdex 200 Increase 10/300 GL column (GE Healthcare, IL, USA) equilibrated with 20 mM sodium phosphate buffer, using the same buffer as the mobile phase at flow rate of 0.5 mL min^−1^. In both cases, proteins of the fractions were monitored at an absorbance of 280 nm. The relative molecular weight (rMW) of proteins were calculated from SDS-PAGE by comparison its relative mobilities to that of the proteins standard.

### Determination of pectinase activity

For semiquantitative determinations, 100 µL of the protein extracellular extracts was deposited into wells cut into agar plates containing 1% w/v pectin with pH of 5.4, 6.2 or 7.0, adjusted with phosphate-citrate buffer. The plates were incubated at 4 °C, 10 °C, 15 °C, 22 °C, 30 °C and 37 °C for 1 to 5 days, and the appearance of a clear halo around the well indicated pectinase activity. For quantitative determinations, the release of reducing sugars from pectin was quantified using the 3-amino-5-nitrosalicylic acid (DNS) method [40]. Briefly, 50 µL of the protein sample was mixed with 50 µl of 10 mg mL^−1^ pectin, incubated at 30 °C for 1 h and then 100 µl of DNS solution was added. After incubation at 100 °C for 10 min, the samples were incubated on ice for 5 min, and the absorbance at 540 nm was measured. For comparative purposes, commercial polygalacturonase was used in activity assays.

### Peptide mass fingerprinting

Protein extracellular extracts were separated by SDS-PAGE and stained with Coomassie Blue G-250. The protein band of interest was cut from the gel and analyzed using the protein analysis service of Alphalyse (Palo Alto, CA, USA). Briefly, the protein sample was reduced and alkylated with carbamidomethylation and subsequently digested with trypsin. The resulting peptides were spotted onto an anchorchip target for analysis on a Bruker Autoflex Speed MALDI-TOF/TOF instrument. The obtained data were analyzed by Mascot, and results having a score greater than 54 (P < 0.05) were considered statistically significant.

### Next-generation Sequencing (NGS)

Cultures of *Tetracladium* sp. were centrifuged at 7000×*g* at 4 °C for 10 min. The cell pellets were used for DNA and RNA purification with the Wizard Genomic DNA Purification Kit (Promega, WI, USA) and RiboMinus Yeast Kit (Thermo Fisher, MA, USA), respectively. The quality and quantity of the samples were determined by absorbance at 260 and 280 nm, and those having a 260/280 ratio of 1.7 to 1.9 and a 260/230 ratio > 2 were used for whole genome sequencing by NGS at Macrogen Inc. (Seoul, South Korea) using the Hiseq 2000 platform.

### Assemblies, ORFs and gene prediction, annotation and expression level analysis

Assemblies were done with ALLPATHS-LG [41]. RNA-seq reads were mapped to the assembled genome with TopHat software [42] and STAR aligner [43]. To capture all junctions, the RNA-seq reads were assembled according to Grabherr et al. [44]. The gene model prediction was made with Augustus [45, 46] and Pasa [47]. Functional annotation was performed using the standard BLAST and InterPro databases. CAZY annotation was made using dbCAN [48] and HMMER3 [49]. ClustalW analysis and gene expression analysis were conducted using Geneious version 10.0.9 [50] and the included plugins.

### Alignment, modeling and bioinformatics analysis

Amino acid sequence alignments were conducted using MEGA7 software [51]. The polygalacturonase sequences chosen for the amino acid sequence comparison had a minimum of 50% similarity and 50% coverage, and corresponded to: *Venturia nashicola* (BAG72101), *Colletotrichum fioriniae* (EXF76863), *Colletotrichum lupini* (ABL01533), *Colletotrichum simmondsii* (KXH46697), *Achaetomium* sp. (AGR51994), *Colletotrichum higginsianum* (XP_018155590), *Venturia pyrina* (BAG72133), *Pestalotiopsis fici* (XP0 07836731), *Talaromyces cellulolyticus* (GAM33350), *Cadophora* sp. (PVH80831), *Pseudomassariellvae xata* (ORY56346).

*Phialocephalasu balpina* (CZR53206), *Penicillium freii* (KUM62405), *Lepidopterella palustris* (OCK84102), *Thielavia arenaria* (AIZ95162), *Fusarium avenaceum* (KIL90067), *Neonectria ditissima* (KPM736 08), *Fusarium venenatum* (CEl70336), *Penicillium griseoroseum* (MF06810), *Colletotrichum gloeosporioides* (ELA24368), *Verticillium alfalfa* (XP0 03002875), *Verticillium dahlia* (XP0 09653008), *Diplodia corticola* (XP_020130427), *Pezoloma ericae* (PMD23778), *Penicillium camemberti* (CRL23357), *Fusarium langsethiae* (KPA37956), *Nectria haematococca* (XP_003040641), *Meliniomyces bicolor* (XP_024742782), *Ustilaginomycotina* sp. (PWN47943), *Talaromycesm arneffei* (KFX46954), *Bipolaris zeicola* (XP_007711986), *Penicillium subrubescens* (OKO96599), *Penicillium brasilianum* (OOQ88122). The polygalacturonase model was constructed using the Swiss-model platform [52], using PDB: 2iq7.1 as the template, which has 91% coverage and 63% identity with the polygalacturonase from *Tetracladium* sp. For validation of the model structure the programs Verify 3D and ERRAT were used trough the AVES v5.0 server (http://servicesn.mbi.ucla.edu/SAVES/), and the values were 96.43% and 89.13%, respectively.

### Heterologous expression of pectinase

The coding sequence of *TPG1* identified in this work (see “Results and discussion” section) was analyzed bioinformatically and modified to generate a sequence of 1066 nt that lacks the first 57 nucleotides, which encode the signal peptide. *Mly* I and *Kpn* I restriction sites were added at the 5′ and 3′ end, respectively, and the codon usage was optimized to the one from *P. pastoris*. The modified gene was synthesized by Genescript company (New York, USA), and the cloning and expression of pectinase was performed using the PichiaPink™ Expression System (Invitrogen, CA, USA) according to manufacturers’ instructions. Briefly, synthetic CDS was ligated to a pPinkα-HC vector and transformed into *E. coli* Top10. The obtained transformants were selected on LB-ampicillin plates, and the presence of recombinant plasmid was confirmed by colony PCR using the primer pair Pectfw/Pectrev (Table 1). Plasmid DNA was purified from selected clones, digested with *Afl*II and transformed into *P. pastoris* PichiaPink strain 2. Several transformants developed on SD plates were selected, and genomic DNA was extracted and checked by PCR using the primer pair Pectfw/Pectrev. Amplicon-positive clones were grown overnight in BCM supplemented with glycerol at 30 °C and then centrifuged at 1500 g for 5 min. Then, the yeast pellets were suspended in 10 ml of BMMY medium and incubated overnight at 30 °C, and methanol was added to reach 4% v/v final concentration. Ten 100 µL culture aliquots were collected at different times from 0 to 24 h and centrifuged at 1.500 g for 5 min, and cellular pellets were suspended in 50 mM sodium phosphate pH 7.4, 1 mM PMSF, 1 mM EDTA and 5% glycerol, and vortexed for 3 min. Samples were directly analyzed by SDS-PAGE.

## Results and discussion

### Screening, selection and identification of secreted pectinases

**Fig. 1:**
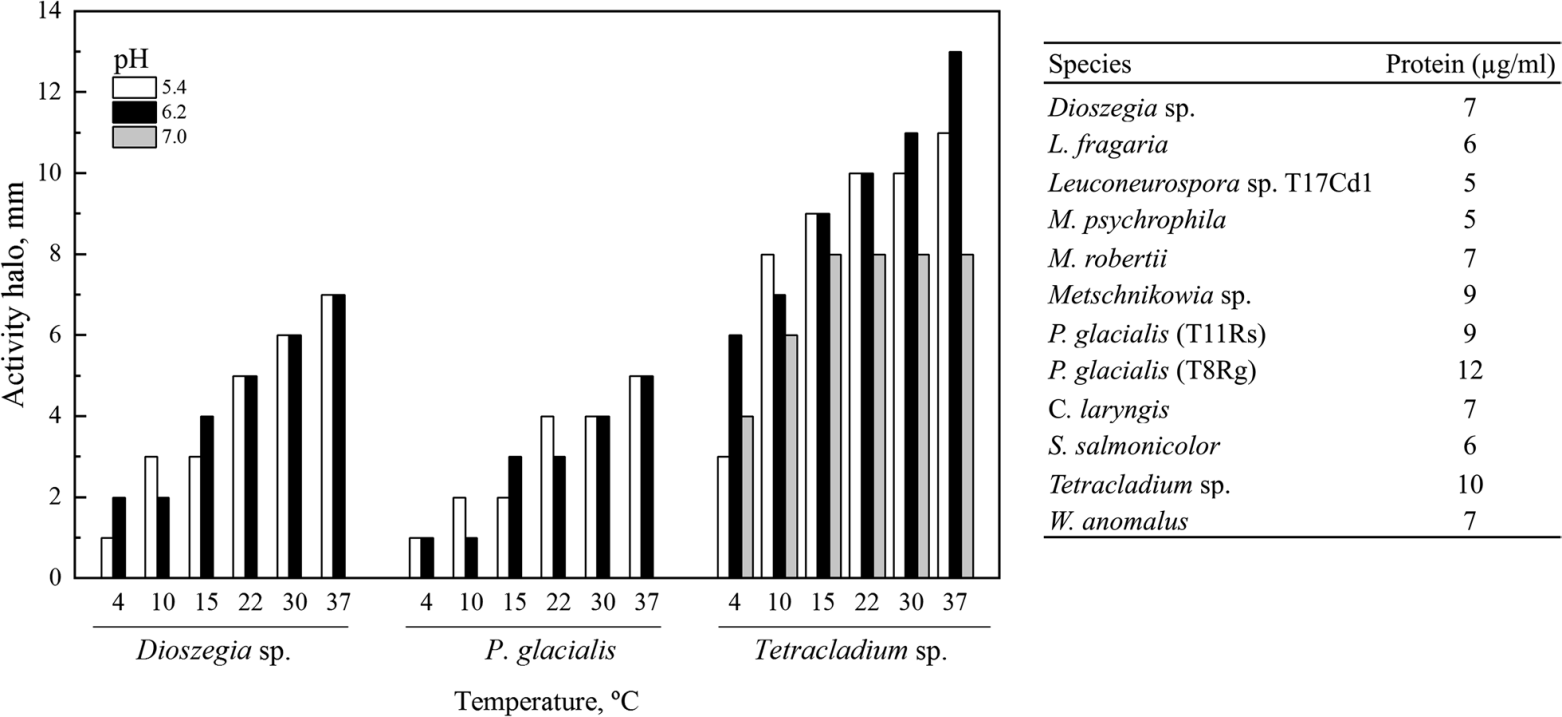
Pectinase activity in plate assays using extracellular proteins secreted by different strains. The activity was determined at 4 to 37 °C and at pH 5.4, 6.2 and 7.0. Only positive results are shown. The total protein concentration of each sample used in the assays is given in the table at the right. The diameter of activity corresponds to the difference between the diameter of the clear halo and the diameter of the well

**Fig. 2:**
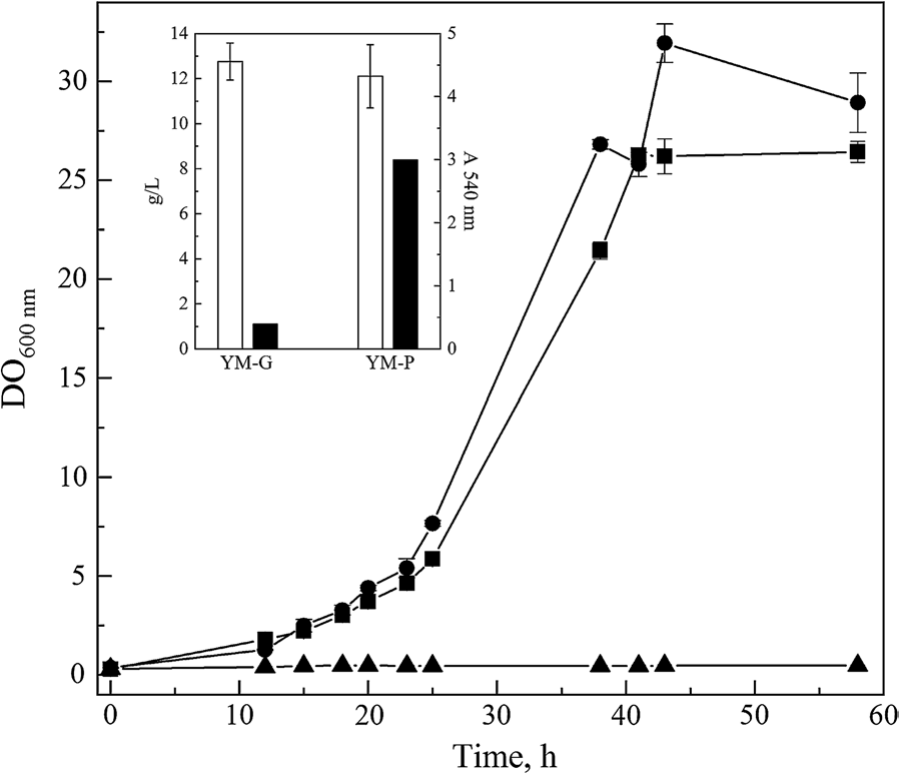
Growth and pectinase activity of *Tetracladium* sp. cultured in different media. The yeast was cultured in YM medium supplemented either with 1% glucose (YM-G, filled circle), 1% pectin (YM-P, filled square) or without supplementation (YM, filled triangle). The inset shows the biomass (white columns) and the pectinase activity (black columns) at the stationary phase of growth. The values are the average of three independent experiments. Data are shown as the average values of three independent cultures

The concentration of protein extracellular extracts obtained from twelve yeasts and yeast-like species cultured in liquid YM medium supplemented with glucose ranged from 5 to 12 µg mL^−1^ (Fig. 1). The obtained protein extracellular extracts were used to test pectin degradation in plates at 4 °C to 37 °C and pH from 5.4 to 7.0, and positive results were observed in three of them (Fig. 1). Pectin degradation was detected from 4 °C to 37 °C and pH from 5.4 and 6.2 in samples from *Dioszegia* sp. and *Phenoliferia glacialis* D7 and from 4 °C to 37 °C and pH from 5.4 and 7.0 in samples from *Tetracladium* sp., which are in the range of described pectinases from other microorganisms [10, 53]. Pectinolytic activity has been previously described in *Rhodotorula mucilaginosa*, *Cystofilobasidium capitatum* [32], *Candida sake*, *Debaryomyces vanrijiae* (now *Schwanniomyces vanrijiae*), *Saccharomyces cerevisiae*, *Candida* sp., *Debaryomyces* sp., *Kluyveromyces marxianus*, *Pichia* sp., *Saccharomyces* sp., *Zygosaccharomyces* sp., [54], *Cryptococcus cylindricus* (now *Piskurozyma cylindrica*), *Mrakia frigida*, *Cystofilobasidium capitatum*, *Cystofilobasidium macerans* and *Rhodotorula mucilaginosa* [55] isolates. In this work, secreted pectinolytic activity is described for the first time in *Dioszegia* sp., *Phenoliferia glacialis* and *Tetracladium* sp. isolates. The highest pectin degradation was observed for protein extracellular extracts from *Tetracladium* sp. at 37 °C and pH 6.2. Therefore, *Tetracladium* sp. was selected for further enzyme purification, identification and heterologous expression. To evaluate if pectin induces the production of pectinase in *Tetracladium* sp., the yeast was grown in YM medium supplemented with glucose or pectin. As shown in Fig. 2, the growth curves were similar in both media, reaching growth rates of 1.2 h^−1^ and 1.1 h^−1^ and maximum biomass of 13 g L^−1^ and 12 g L^−1^ in medium supplemented with glucose and pectin, respectively. However, extracellular pectinase activity at the initial stationary phase of growth was almost seven-fold higher in the medium supplemented with pectin than with glucose, indicative of a induction of the production of pectinolytic enzymes in presence of pectin. Similarly, an induction effect of pectin in the production of endo-PG in *Aspergillus niger* T0005007-2 was observed [56].

**Fig. 3:**
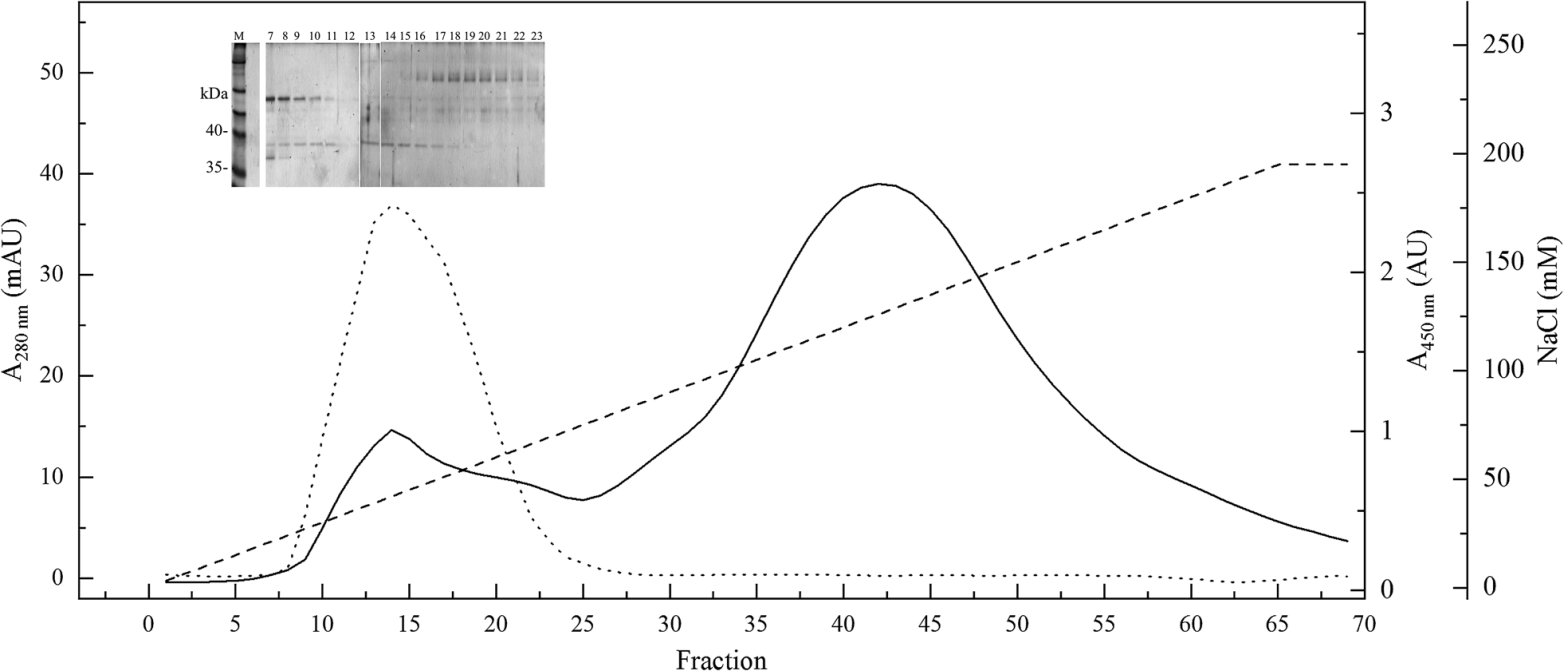
Protein purification by ionic exchange chromatography. Purification profile of the 80% ammonium sulfate fraction from *Tetracladium* sp. culture supernatant is shown. A NaCl gradient from 3 to 195 mM was used as the mobile phase (discontinuous line), and protein presence was detected by absorbance at 280 nm of the fractions (continuous line). Pectinase activity in each fraction was determined (dotted line), and protein content was evaluated by SDS-PAGE (inserted gel photo). SDS-PAGE lanes coincide with the corresponding fraction in the chromatogram. The arrow indicates the probable pectinase enzyme. M, protein marker; E, sample of 80% ammonium sulfate

To purify the pectinase enzyme, *Tetracladium* sp. was cultivated in YM medium supplemented with pectin, and culture supernatant proteins were fractioned using increasing saturation of ammonium sulfate (20 to 80%). Pectinase activity was detected in fractions corresponding to 40, 60 and 80% ammonium sulfate having 7, 33 and 60% total activity, respectively. The protein sample corresponding to 80% ammonium sulfate precipitation was separated by ionic exchange chromatography (Fig. 3), and two main peaks (fractions 14 and 42) were obtained; pectinase activity was detected in fractions surrounding fraction 14. According to the SDS-PAGE analysis of these fractions, the pectinase activity was associated with a protein band of rMW 38,000 (arrow in Fig. 3), which was purified, concentrated and analyzed by peptide mass fingerprinting (PMFP). The MW of described microbial polygalacturonases is widely variable ranging from 6500 to 320,000 according to the BRENDA enzyme database [57]. From the analysis of the results and comparison using the Mascot database search engine, it was found that peptides ARAACTFSGATG, IKNSDNGVRIKTIEG, DIVYKDITLVNIA and KVTMNNVAGDTKGGHNTDAF matched with 91, 87, 77 and 79% identity with polygalacturonases described from *Fusarium avenaceum* (KIL93978.1), *Sclerotinia sclerotiorum* (CAA74019.1), *Ascochyta rabiei* (KZM23605.1) and *A. niger* CBS 513.88 (XP_001397067.1), respectively.

### Identification of the putative pectinase-encoding gene

**Fig. 4:**
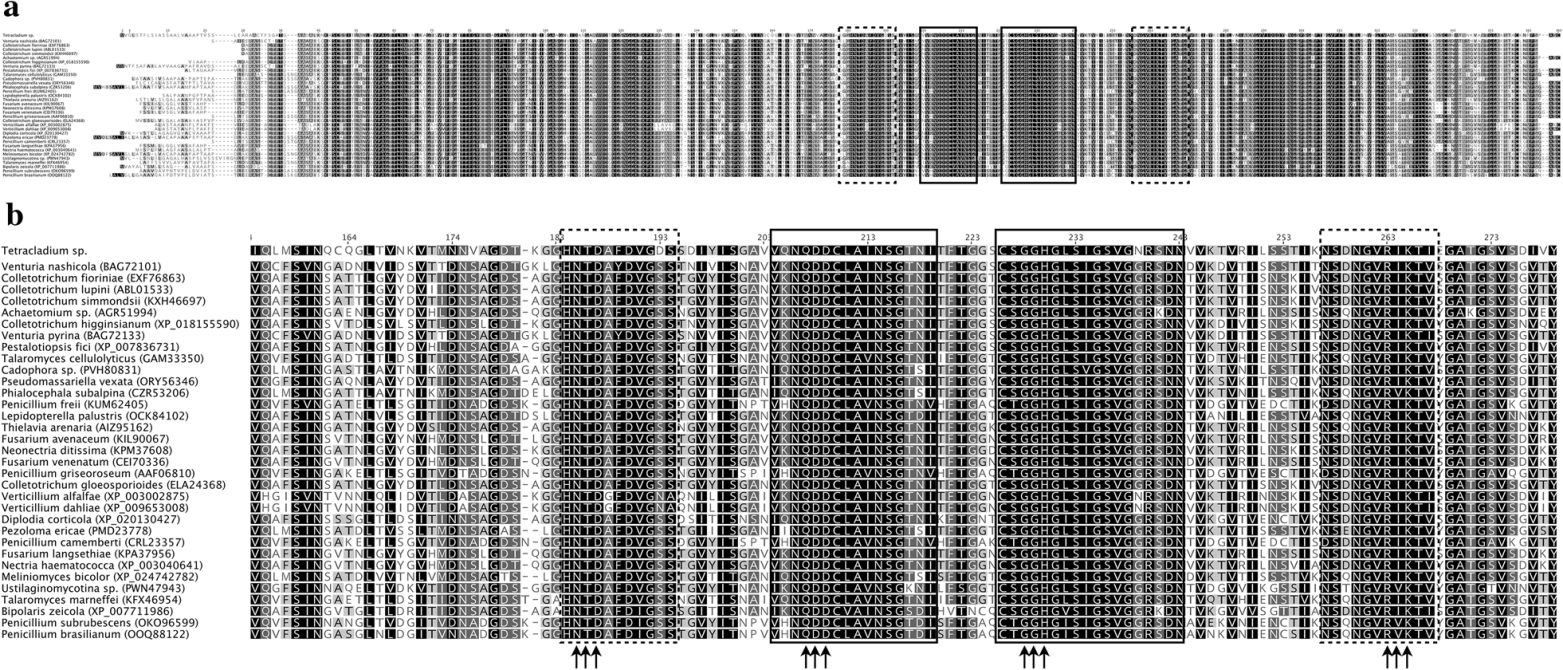
Alignment of fungal polygalacturonases primary structures. Global (**a**) and detailed (**b**) comparison of fungal polygalacturonases. The conserved regions corresponding to the active site and to the substrate binding site are enclosed in continuous and discontinuous squares, respectively. The conserved residues in each region are indicated by arrows

**Fig. 5:**
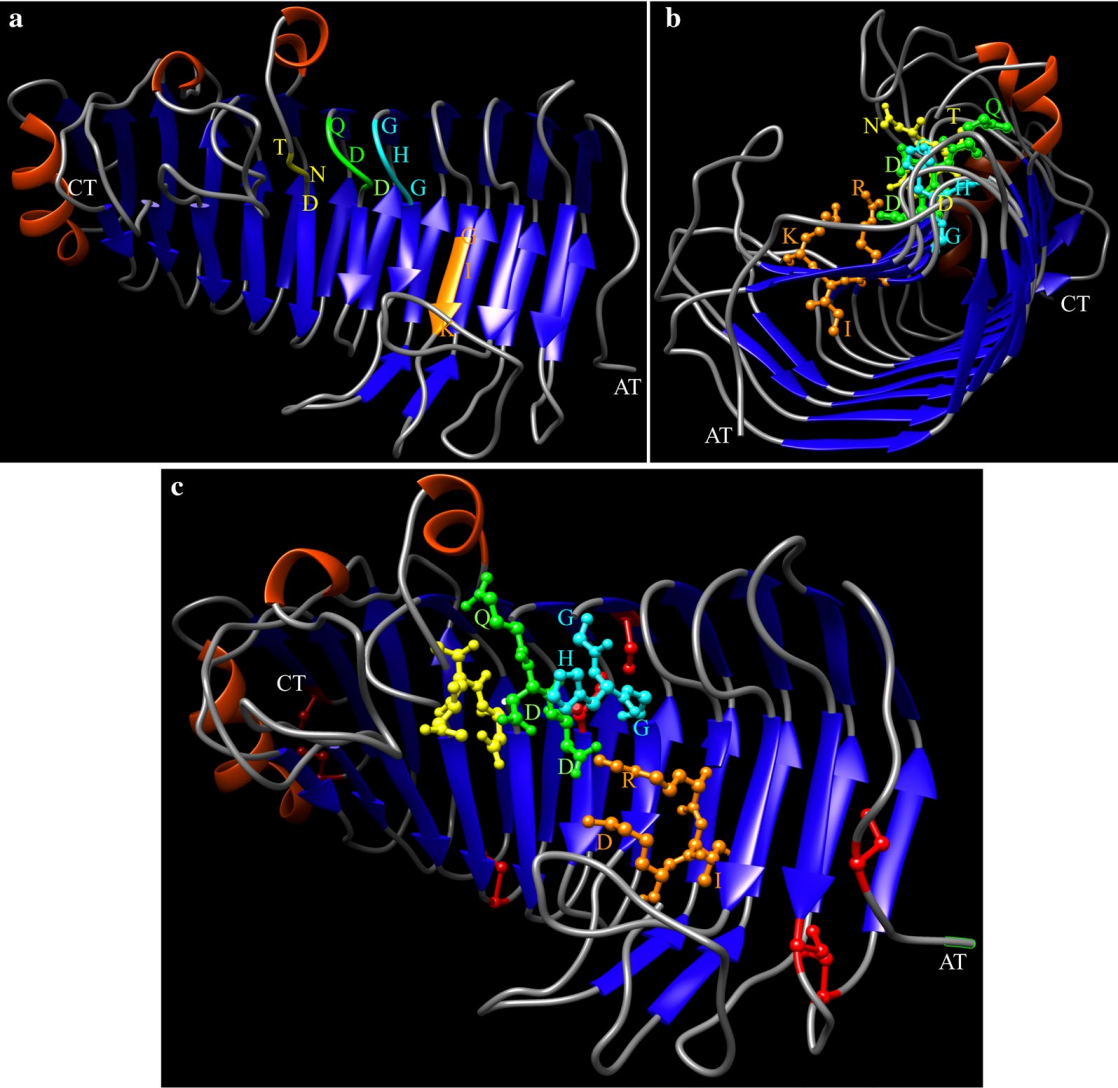
3D structure model of TPG1. In** a** and** c**, the N-terminus of the protein is on the right and in** b**, it is in front. Conserved motifs are shown in color and/or in balls-and-sticks: catalytic residues QDD are in green and GHG in blue; substrate binding residues RIK are in orange and NTD in yellow. Conserved cysteine residues are shown in red balls-and-sticks in **c**. AT, amino terminus; CT, carboxyl terminus

The putative CDS were predicted in the genomics scaffolds of *Tetracladium* sp. and its transcriptomes, when grown in YM medium supplemented with glucose or pectin were determined. The ORFs predicted in the transcriptomes were mapped to genomic scaffolds, and those having 100% identity and a correct exon–intron structure were considered for comparison to the NCBI database and annotation. Thus, 7 putative polygalacturonase genes (lengths from 1325 to 4827 bp) and 14 putative pectate lyase genes (lengths from 993 to 2712 bp) were found. The expression at the transcript level of these putative genes was determined in both culture conditions (Table 2). The majority of the putative pectate lyase-encoding genes did not show significant differences in the expression level between both culture conditions, except for scaffold_2.g849.t1 and scaffold_3.g812.t1, which had a 28- and 112-fold expression increase respectively, in cultures supplemented with pectin in comparison to glucose. In contrast, all the putative polygalacturonase-encoding genes increased their expression in medium supplemented with pectin compared to glucose, with scaffold_10.g413.t1 (12,228-fold) and scaffold_6.g590.t1 (12,360-fold) having the highest increases. Similar to these results, a higher transcript level of gene *pgg1* that encodes a pectinase in *Penicillium griseoroseum* was observed in the presence of pectin with in comparison to without yeast extract [58]. As mentioned above concerning the analysis by PMFP of the purified pectinolytic enzyme, it was found four peptides that matched with polygalacturonases from other fungi. These peptides were compared to the translated sequences of all of the putative CDS for polygalacturonases and pectate lyases identified in *Tetracladium* sp., found that all peptides mapped only with translated sequence from a CDS named scaffold_10.g413.t1. Is important to mention that along with our purification methodologies the focus was in finding the enzyme having the highest activity; therefore the other CDSs may encode for putative pectinolytic enzymes with a minor activity that were not considered in this work. Therefore, the enzyme purified from *Tetracladium* sp. is a polygalacturonase and the encoding gene was named *TPG*1, which is composed of 5 exons and encodes a protein of 368 residues (Additional file 1: Fig. S1). Figure 4 shows the alignment of the translated sequence from *TPG*1 and pectinases described in other fungi. Globally, there are several conserved residues (Fig. 4a), including those in the active and substrate binding sites (Fig. 4b) [59]. A three-dimensional model of the *Tetracladium* sp. TPG1 was constructed (Fig. 5) using the crystal structure of the endopolygalacturonase from the phytopathogenic fungus *Colletotrichum lupini* (CluPG1) as the template [60]. The overall predicted structure of TPG1 corresponded to a right-handed beta-helical structure that forms a large cleft (Fig. 5b), which is a common feature in other pectinases, such as endo-polygalacturonases from *Fusarium moniliforme* [61] and from *A. niger* [62, 63], and polygalacturonases from *Ewinia carotovora* [64], *Aspergillus aculeatus* [65], and *C. lupini* [60]. The putative catalytic residues in TPG1 corresponded to QDD and GHG and the substrate binding residues to RIK and NTD (Fig. 5). Furthermore, there were conserved cysteines (Fig. 5c), which are important for disulfide bridge formation, especially in fungal polygalacturonases [59].

**Table 2:** Expression, at the transcript level, of putative pectate lyase- and polygalacturonase-encoding genes from *Tetracladium* sp

Putative	CDS	Expression (FPKM)		P/G^a^
		YM-G	YM-P	
PL	scaffold_4.g450.t1	3.6	0.8	− 4
PL	scaffold_5.g260.t1	22.9	6.5	− 4
PL	scaffold_4.g243.t1	2.2	0.8	− 3
PL	scaffold_2.g1041.t1	2.4	0.9	− 3
PL	scaffold_0.g2020.t1	5.6	3.3	− 2
PL	scaffold_1.g759.t1	6.5	4.2	− 2
PL	scaffold_1.g52.t1	29.9	22.0	− 1
PL	scaffold_0.g2289.t1	1.1	0.8	− 1
PL	scaffold_11.g858.t1	26.0	45.2	2
PL	scaffold_7.g463.t1	7.0	15.0	2
PL	scaffold_3.g58.t1	18.0	60.9	3
PL	scaffold_0.g2293.t1	5.5	19.7	4
PG	scaffold_3.g273.t1	1.6	11.8	7
PG	scaffold_3.g424.t1	19.1	243.4	13
PL	scaffold_2.g849.t1	2.6	74.9	28
PG	scaffold_3.g812.t1	0.6	66.3	112
PL	scaffold_3.g812.t1	0.6	66.3	112
PG	scaffold_0.g606.t1	3.9	1012.9	258
PG	scaffold_3.g1104.t1	1.0	291.1	288
PG	scaffold_10.g413.t1	0.1	1762.5	12,228
PG	scaffold_6.g590.t1	0.0	566.6	12,360

Expression in medium supplemented with glucose (YM-G) or pectin (YM-P). *PL* pectate lyase, *PG* polygalacturonase. ^a^Expression fold changes between cultures in YM-P (P) and YM-G (G) media

### Heterologous expression and characterization of *TPG*1

**Fig. 6:**
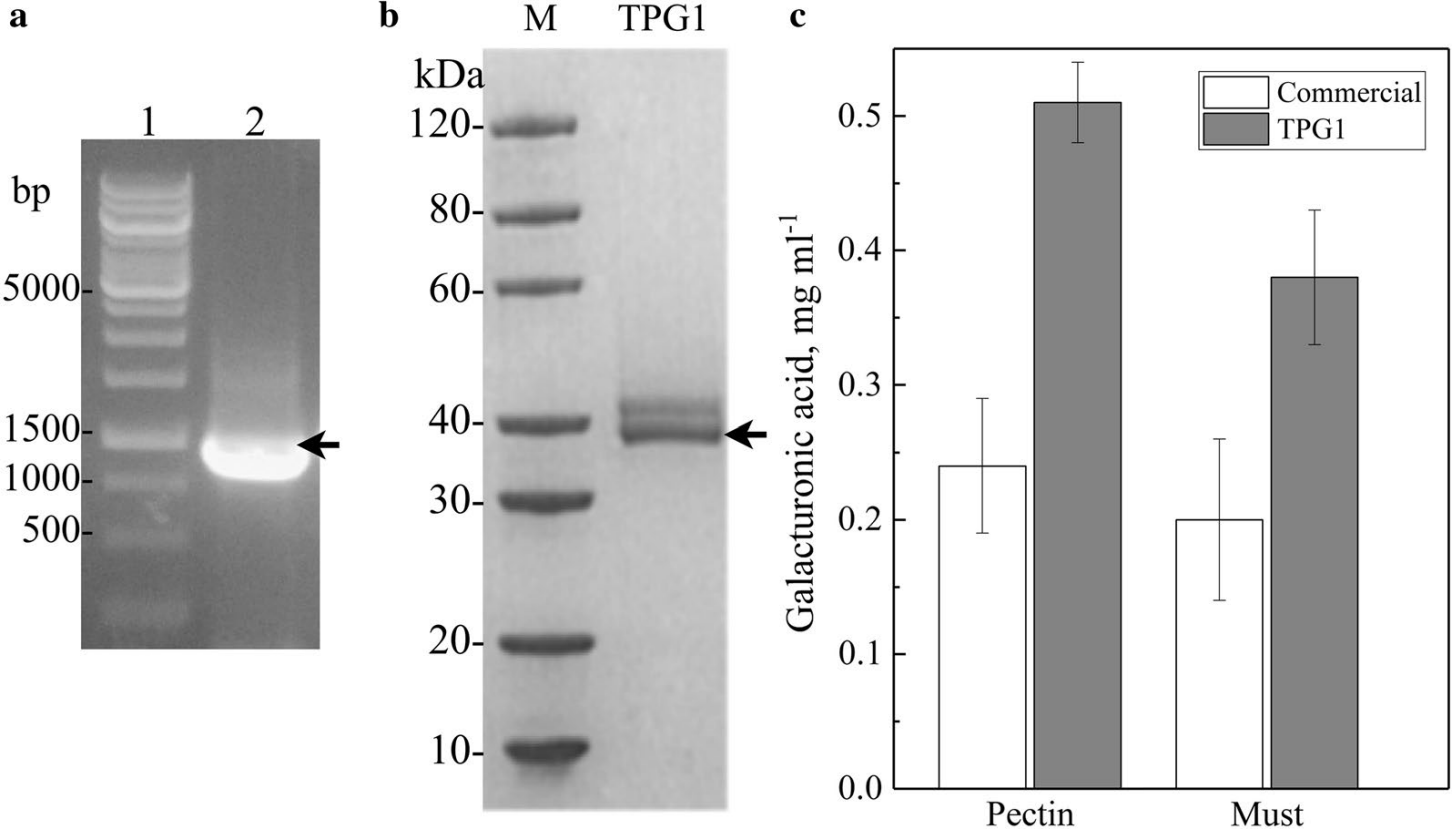
Characterization of the recombinant TPG1. **a** Analysis by PCR of a recombinant clone (lane 2) in which an amplicon with a size expected for synthetic *TPG1* is observed (arrow); Lane 1, DNA ladder. **b** SDS-PAGE. The arrow indicates the protein band corresponding to the recombinant TPG1; M, molecular marker. **c** Pectinase activity of TPG1 and a commercial polygalacturonase, evaluated at pH 3.0 and 15 °C using pectin or must as substrate. Data shown are the average values of three independent enzymatic assays

The synthetic sequence of *TPG1* was inserted in the pPinkα-HC vector and trasformed in *P. pastoris* strain PichiaPink. Clones were analyzed by colony PCR using the primers pair Pectfw and Pectrev, the plasmid DNA was purified from one of the clones showing amplicons of the expected length and PCR-amplified with the same primers pair, obtaining an amplicon with a size expected for synthetic *TPG1* (Fig. 6a). The selected clon was cultured in BMGY medium at 30 °C for 24 h, then the production of pectinase was induced by changing the medium to BMMY and incubation at the same temperature for 6 to 72 h. Extracellular proteins were obtained from cultures and analyzed by SDS-PAGE observing an intense protein band of approximately 38,000 rMW (Fig. 6b). The recombinant TPG1 and a commercial polygalacturonase were used in pectinase activity assays, using purified pectin and wine must as substrates; the hydrolysis of pectin was evaluated as the liberation of glucuronic acid at 15 °C and pH 3.0 in 1 h. The amount of glucuronic acid released by the recombinant TPG1 was twofold higher than the amount released by the commercial polygalacturonase using either substrate (Fig. 6c).

## Conclusion

The polygalacturonase TPG1 was identified in *Tetracladium* sp., and its expression, determined at the transcriptional and enzyme activity levels, is induced by pectin. The TPG1-encoding gene was successfully expressed in *P. pastoris*, and a recombinant polygalacturonase highly active at 15 °C was obtained. Furthermore, the feasibility to cultivate *P. pastoris* in high-cell density fermentations facilitates de the efficient production of pectinase at amount and purity required to be applied in industrial processes such as clarification processes of fermented beverage and fruit juice production. Therefore, the recombinant pectinase described in this work is economically attractive by both its efficient production and its activity at low temperatures allowing energy-saving in the processes.

## Additional file

**Additional file 1: Fig. S1.** TG1 sequence and gene structure.
