# Supplementary figures and images for: Pectinase secreted by psychrotolerant fungi: identification, molecular characterization and heterologous expression of a cold-active polygalacturonase from Tetracladium sp
Source: Microb Cell Fact. 2019 Mar 7;18:45. doi: 10.1186/s12934-019-1092-2 (PMC6407229; doi:10.1186/s12934-019-1092-2)

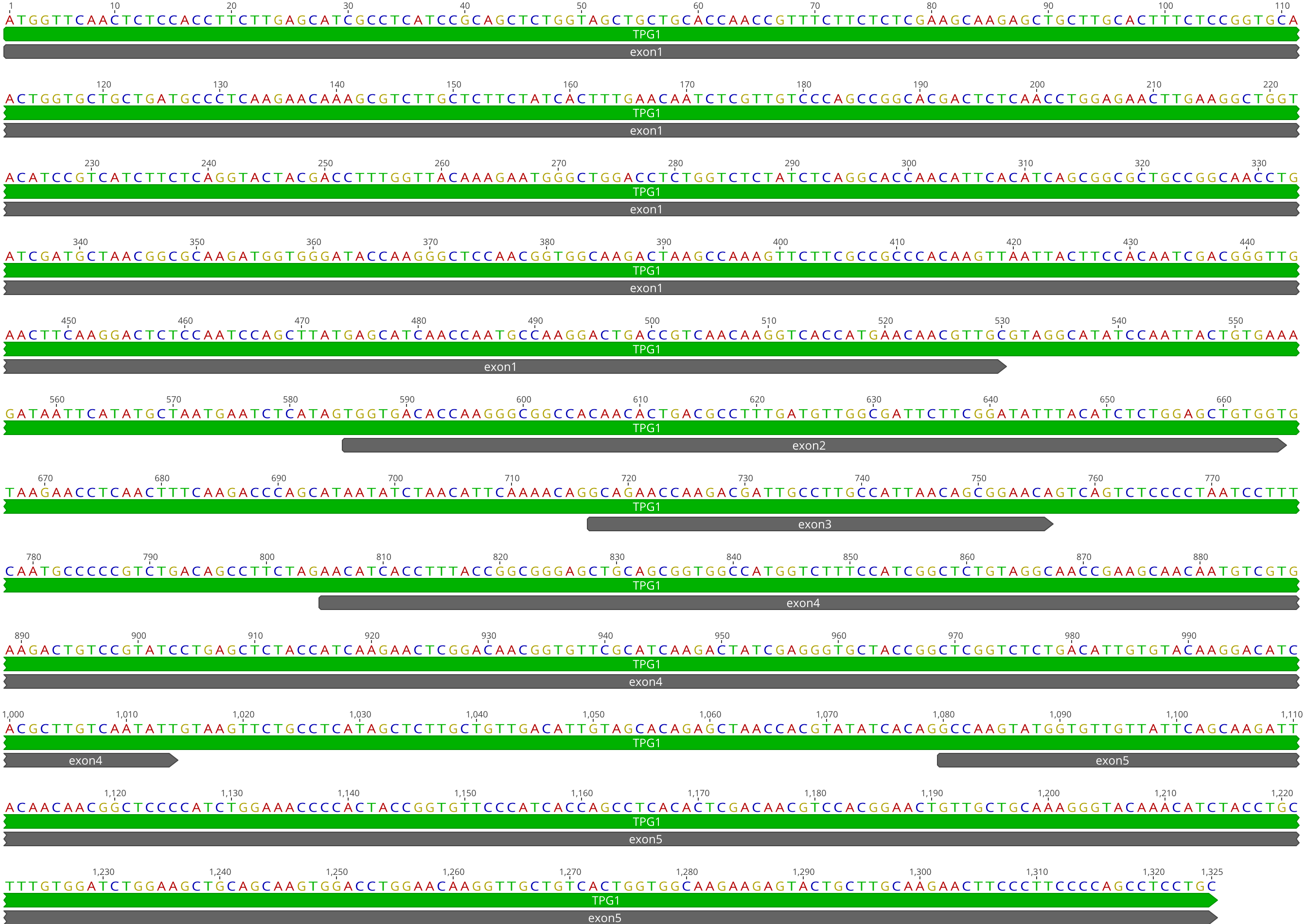

Supplement: Supplementary file 1 — Additional file 1: Fig. S1. TG1 sequence and gene structure. [file 12934_2019_1092_MOESM1_ESM.pdf]
